# Escape rate and diffusion of a Stochastically Driven particle

**DOI:** 10.1038/srep41442

**Published:** 2017-01-25

**Authors:** Antonio Piscitelli, Massimo Pica Ciamarra

**Affiliations:** 1 Division of Physics and Applied Physics, School of Physical and Mathematical Sciences, Nanyang Technological University, Singapore, Singapore; 2 CNR–SPIN, Dipartimento di Scienze Fisiche, Università di Napoli Federico II, I-80126, Napoli, Italy

## Abstract

The dynamical properties of a tracer repeatedly colliding with heat bath particles can be described within a Langevin framework provided that the tracer is more massive than the bath particles, and that the collisions are frequent. Here we consider the escape of a particle from a potential well, and the diffusion coefficient in a periodic potential, without making these assumptions. We have thus investigated the dynamical properties of a Stochastically Driven particle that moves under the influence of the confining potential in between successive collisions with the heat bath. In the overdamped limit, both the escape rate and the diffusion coefficient coincide with those of a Langevin particle. Conversely, in the underdamped limit the two dynamics have a different temperature dependence. In particular, at low temperature the Stochastically Driven particle has a smaller escape rate, but a larger diffusion coefficient.

The evaluation of the rate of escape of a particle from a one dimensional potential well, and of the diffusion coefficient of a particle moving in a one dimensional periodic potential, are classical problems in statistical physics that are relevant to the physical, chemical, engineering and biological sciences. When the timescale of interaction of the particle with the heat bath is the smallest timescale of the problem^1^, escape and diffusion can be investigated within a Langevin formalism. In this context, solutions have been obtained^2^ in both the overdamped, 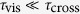, and underdamped limits, 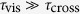. Here *τ*_vis_ = *m/γ* is the viscous relaxation timescale, with *m* mass of the particle and *γ* the viscous friction coefficient, and *τ*_cross_ = 1/*ω*_*b*_ is a timescale related to the exchange between kinetic and potential energy during barrier crossing fixed by the shape of the potential *V(x*) on the top of the barrier, 
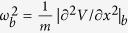
. In a variety of different contexts including research strategies in biology^3^, transport in electronics^4^, market evolution models^5^, supercooled liquids^6^,^7^,^8^, diffusion of atoms in optical lattices^9^, diffusion of molecules at liquid/solid interfaces^10^, chemical reaction rates^11^,^12^, the time scale of interaction with the heat bath is not the smallest one, and the Langevin approach is no longer justified. In these cases one should adopt a different formalism, allowing the particle to move under the influence of the potential in between successive collisions with the heat bath.

Here we report on the first investigation of escape and diffusion problems within this formalism. We consider a simple model in which a walker interacts with the heat bath with a constant rate *t*_*c*_, the interactions instantaneously randomizing the walker’s velocity according to the Maxwell-Boltzmann distribution at the considered temperature. When not interacting with the heat bath, the walker moves according to Newton’s equation within the potential. This model has been investigated before to describe chemical reaction rates. In this context *t*_*c*_ depends on the pressure of the gas surrounding the walker, and the walker and the surrounding ones have the same mass^11^,^12^. The model has also been generalized considering the general case of different masses^13^, in which case the Klein-Kramers’ equation is recovered as a limit of frequent collisions and large walker mass^14^, as well as considering how anomalous diffusion could be related to the distribution of the collision intertimes^15^. In particluar, despite Lévy flights in periodic potentials have attracted some attention^16^,^17^, the escape properties in the presence of an intertime distribution with finite moments has not yet been studied systematically.

As a model potential, we have considered a periodic *x*^4^ well, *V(x*), with period *L* and energy barrier *V*(±*L*/2) = Δ*U*, but our results are easily generalizable. Thus in the period –*L*/2 ≤ *x* ≤ *L*/2, the potential is 
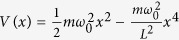
. The energy barrier is 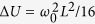, while 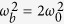. The tracer particle collides elastically with the bath particles, that have the same mass. This implies that in a collision the tracer and the bath particle exchange their velocity. Thus, the noise acts as follows: at exponentially distributed time intervals 
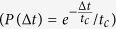
 the tracer undergoes a collision, meaning that its velocity is instantaneously re-sampled from a Gaussian distribution with variance equal to the temperature 
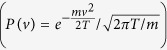
. The simulation procedure is detailed in the *Methods* section.

We have determined the escape rate and the diffusion coefficient in both the overdamped, 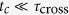, and the underdamped limits, 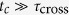, validating our theoretical results against numerical simulations and highlighting qualitative and quantitative differences with respect to the Langevin dynamics. In Fig. 1 we illustrate typical trajectories of the Stochastically Driven and of the Langevin dynamics, in the overdamped and the underdamped limits. In both cases the trajectories of the two dynamics appear qualitatively similar. However, we will show that only in the overdamped limit the two dynamics quantitatively agree. Indeed, in the underdamped limit the two dynamics differ, the Langevin one having an higher escape rate but, surprisingly, a smaller diffusion coefficient. This clarifies that the Brownian model is not appropriate in the underdamped limit to describe a physical system where the interaction with the heat bath occurs via successive collisions rather than via a continuous interaction.

## Results

### Escape rate

The escape rate is conventionally defined as the rate with which a particle “irreversibly” escapes from a well in a given direction. In a one dimensional periodic potential, the notion of irreversibility is easily clarified. A barrier crossing event is irreversible if it is not correlated to the subsequent one. Thus, an irreversible barrier crossing event is followed with probability 1/2 by a barrier crossing that brings the particle back to its original well, and with probability 1/2 by a barrier crossing event that brings the particle to the following well. To estimate the escape rate, we consider that the physical process leading to an irreversible escape comprises different steps. First, starting from thermal equilibrium, a particle performs a barrier crossing jump entering the arrival well. We indicate with 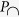 its probabily. Then, the particle performs different jumps remaining localized close to the top of the barrier, possibly crossing the top of the barrier a number of times. We indicate with *p* the probability that the particles crosses the barrier an even number of times, so that it remains in the arrival well. Finally, the particles moves away from the top of the barrier decorrelating in the arrival well, without the occurrence of any further recrossing. We call *P*_d_ this probability. The escape rate is then given by 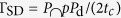. Since the probability that a particle recrosses a barrier an even number of times is 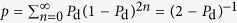, one finally gets


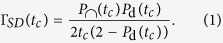


To estimate the escape rate, we thus need to estimate the barrier crossing probability 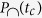, and the decorrelating probability *P*_d_. 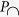 is obtained from an equilibrium average over the jumps. Indeed, each jump is characterized by three variables, the coordinate of the starting point, *x*_*s*_, the initial velocity *v*_*s*_, and the time of flight *t. x*_*s*_ and *v*_*s*_ have a Boltzmann and Maxwellian equilibrium distribution, respectively, while *t* is exponentially distributed with time constant *t*_*c*_. Alternatively, each jump can be characterized by *x*_*s*_, by the coordinate of the final point, *x*_*e*_, and by the total energy *E*. Assuming with no loss of generality |*x*_*s*_| < *L*/2, 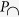 is the probability that |*x*_*e*_| > *L*/2, which is found to be 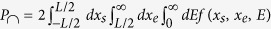, where


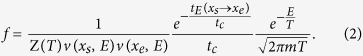


**Figure 2:**
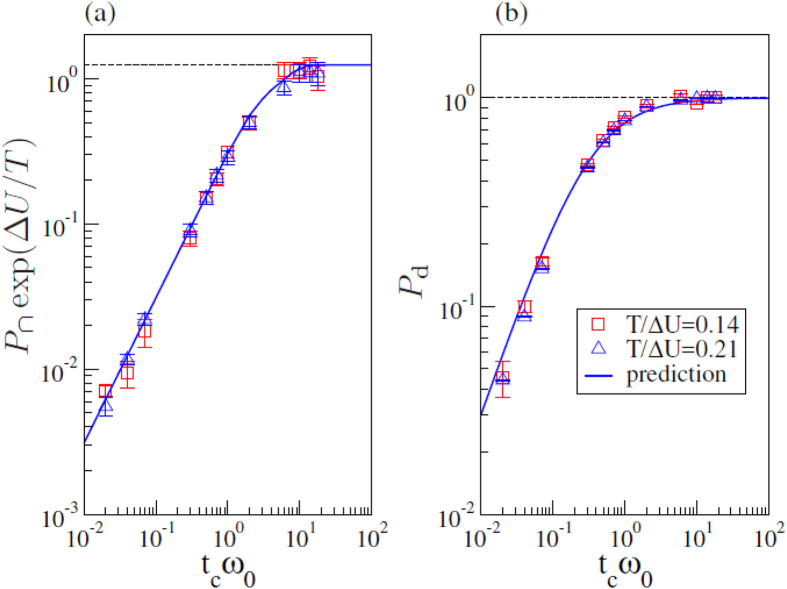
(**a**) Probability 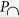 that a jump crosses an energy barrier, normalized with the Arrhenius factor. The full line is our theoretical prediction, the dashed line the asymptotic value for Δ*T/U* = 0.21. (**b**) Fraction of uncorrelated barrier crossing jumps, *P*_d_. The full line is an empirical fitting formula based on the predicted behavior in the *t*_*c*_ → 0, ∞ limits (see text).

Here *f (x*_*s*_, *x*_*e*_, *E*) is the probability that the walker interacts with the heat bath when in position *x*_*s*_, that through this interaction it acquires a total energy *E*, and that its flight time equals the time needed to travel from *x*_*s*_ to *x*_*e*_ with total energy *E*, which is given by 
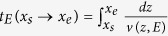
. 
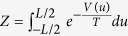
 is a temperature dependent normalization constant. The predicted dependence of 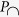 on *t*_*c*_ illustrated in Fig. 2a (full line) correctly describes the numerical results. The high and small *t*_*c*_ limits can be rationalized. In the *t*_*c*_ → 0 limit, the above triple integral can be carried out, and one finds 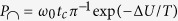. In the *t*_*c*_ → ∞ limit all jumps with enough energy cross the barrier and 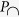 approaches a constant, whose weak temperature dependence is neglected in the following.

The decorrelation probability *P*_d_ is estimated considering that, if a particle reaches a position which is at a far enough distance *l*_*T*_ from the top of the barrier, then it decorrelates as its dynamics becomes dominated by the potential. We assume *l*_*T*_ to be the distance at which the potential significantly affects the velocity 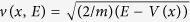 of a particle crossing the barrier with energy *E*, and estimate 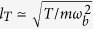 through a Taylor expansion of *v(x, E*) around the top of an energy barrier. In the overdamped limit 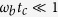, *P*_d_ is given by the probability that a barrier crossing event is followed by a sequence of jumps (of typical size ∝ 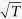) able to drive the particle at distance 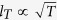 from the top, before a recrossing occurs. It is easy to show in a mean first passage time formalism^18^ that in this regime 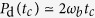. In the underdamped limit, 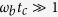, jumps are long and barrier crossing events can be considered irreversible, so that *P*_d_ = 1. The numerical measure of *P*_d_(*t*_*c*_) confirms our predictions for the overdamped and underdamped limits of *P*_d_, as illustrated in Fig. 2b. We approximate in the following *P*_d_ with a simple functional form able to capture the crossover between the 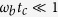 and the 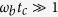 limits, 
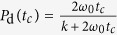
. We fix *k* = *ω*_0_/*ω*_*b*_ exploiting an analogy with the Langevin dynamics in the overdamped low temperature limit, we will detail below.

**Figure 3:**
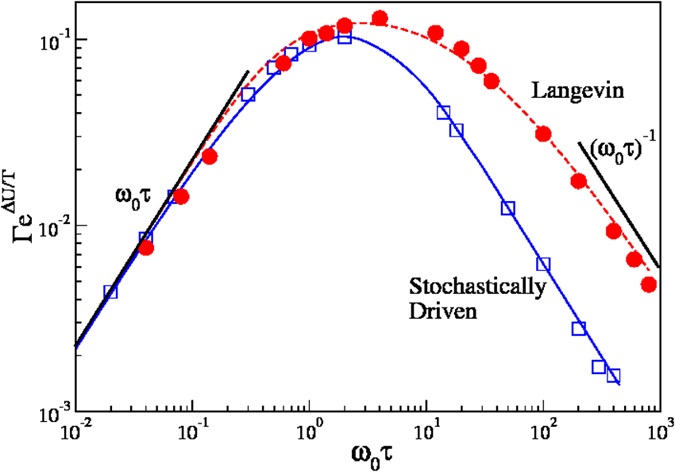
Numerical results and theoretical predictions for dependence of the jump rate on τ = t_c_ = τ_vis_, for the Stochastically Driven dynamics and the Langevin dynamics. The temperature is *T*/Δ*U* = 0.21. The full line is our theoretical prediction for 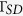. The dashed line is an empirical expression that interpolates between the overdamped and underdamped limits of 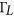 (Eq. 6.4 of ref. 2).

Having determined 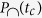 and *P*_d_(*t*_*c*_), we can compute the escape rate 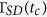 at all *t*_*c*_ through Eq. 1. The overdamped and the underdamped limits result 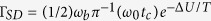 and 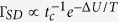, respectively. Figure 3 shows that our theoretical prediction (full line) well compares with numerical simulations of the model (open squares), at all *t*_*c*_. In the figure, we also illustrate numerical results (full circles) for the escape rate of the Langevin dynamics, 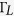. We remind that in the medium/high damping regime, and in the low temperature limit, 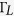 is the celebrated Kramers’ escape rate^19^, 
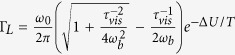
, and that finite temperature corrections have been determined by Lifson–Jackson^20^. In the overdamped limit, Kramer’s result coincides with our prediction for 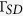 provided that *k* = *ω*_0_/*ω*_*b*_ in our functional form for *P*_d_. In the underdamped limit, 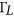 is known to scale as 
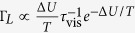
2. This result clarifies that, as concern the escape rate, the two dynamics markedly differ in the underdamped limit. The Langevin dynamics has a much higher escape rate, being 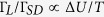.

### Diffusivity

**Figure 4:**
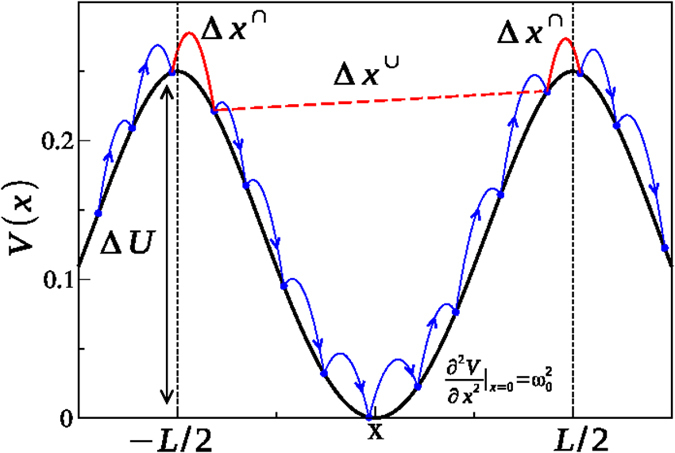
Schematic representation of the definitions of the internal and external jumps in the Stochastically Driven particle. The height of the barrier Δ*U*, the period *L* and the curvature in the minimum 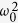 are indicated.

The key idea that allows to obtain analytical results for the diffusivity of a stochasitcally driven particle in a confining potential is the introduction of the coarse-grained trajectory illustrated in Fig. 4. Indeed, each trajectory can be conveniently described as a sequence of barrier crossing jumps, with displacement 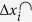, followed by effective intra-well jumps, with displacement 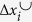. The effective displacement 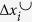 is the total displacement of the intra–well jumps connecting the final position of jump 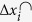, and the initial position of jump 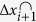. Since the fraction of barrier-crossing jumps is 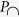, after 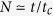 jumps the overall displacement is 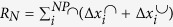, and the diffusion coefficient is


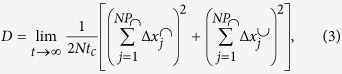


the cross product term vanishing for symmetry reasons. Since only a fraction *P*_d_ of the terms appearing in the above sums are uncorrelated, the diffusion coefficient can also be expressed as


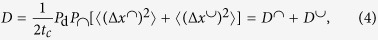


where 〈·〉 indicates *averages over uncorrelated jumps*. Thus, we are left with the problem of estimating the mean square jump length of uncorrelated barrier crossing jumps, 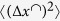, and of uncorrelated effective intra-well jumps, 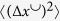.

In the overdamped *t*_*c*_ → 0 limit all jumps are short, and barrier crossing jumps start and end close to the top of a barrier, where the potential is flat. Accordingly, 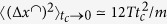
21, and given our results for *P*_d_ and 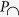,


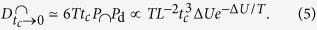


To estimate 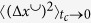 we consider that, since barrier crossing jumps are short, two subsequent barrier crossing jumps are connected by a sequence of jumps whose total displacement is either zero, if the particle recrosses the same barrier, or roughly equal to the period of the potential, if the particle traverses a well and crosses a subsequent barrier. For uncorrelated barrier crossing jumps these two possibilities are equally likely, which implies 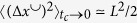. This allows to estimate


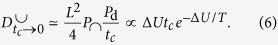


In the underdamped *t*_*c*_ → ∞ limit a particle that has enough energy to cross an energy barrier will traverse 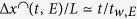 wells, where *t* is the jump duration, and *t*_*w,E*_ the time the particle needs to cross a single energy well. Thus 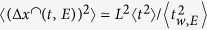 is evaluated averaging over the waiting time distribution 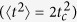 and over the energy of the particle. This leads to 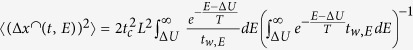, that scales as


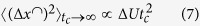


since 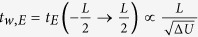 for small *E* − Δ*U*. We thus estimate


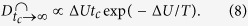


To determine 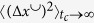, we indicate with *p*_*k*_ the probability that a walker interacts *k* times with the heat bath in a well, before escaping. If *x*_*e*_ is the original position inside the well, and *x*_*s*_ the final one, then 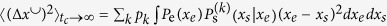. Here 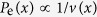 is the probability that a barrier crossing jump ends in *x*_*e*_, one could evaluate from the equilibrium distributions over the barrier-crossing jumps, and 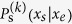 is the probability that the jump through which the particle escapes from the well starts in *x*_*s*_, being the particle arrived in *x*_*e*_. To a good approximationa particle exits from the well after performing a single collision, so that *x*_*e*_ − *x*_*s*_ = 0, or after thermalizing within the well, so that *x*_*e*_ and *x*_*s*_ becomes uncorrelated. Accordingly, 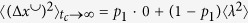, where


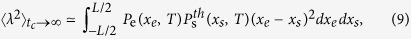


with 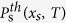 the probability that a barrier crossing jumps of a thermalized state starts from position *x*_*s*_. The evaluation of both *p*_1_, *P*_e_(*x*_*e*_, *T*) and 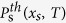 leads to 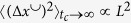. Summarizing, in the *t*_*c*_ → ∞ limit


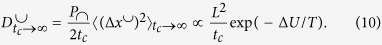


We finally note that, in both Eqs 8 and 10, the proportionality constants have a weak temperature dependence we neglect, that is fixed by the shape of the potential.

**Figure 5:**
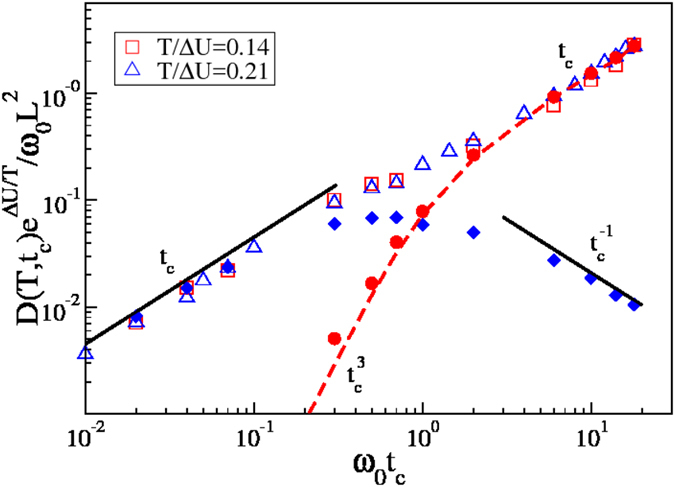
Open symbols illustrate the dependence of the normalized diffusion coefficient on the typical time of collision with the heat bath at low temperatures. Full symbols indicate the contribution due to 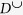 (diamonds) and 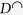 (circles). Full lines are predictions for 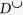 in the overdamped and in the underdamped limits, while the dashed line is the analytical prediction of 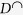, at all *t*_*c*_. All predictions are for *T*/Δ*U* = 0.21.

While we have estimated 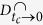 and 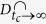 in the low temperature regime, it is also possible to estimate 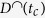 at all *t*_*c*_. To this end we assume the barrier crossing jumps to be always uncorrelated, which is reasonable as the jumps are uncorrelated both in the overdamped limit, as jumps are short and particles on the top of the barrier move as free particles, and in the underdamped limit. With this assumption we estimate 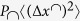 from equilibrium average over the jumps, 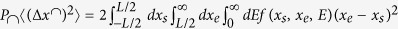, with *f* given in Eq. 2, and thus get 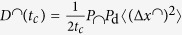. Beside being valid at all *t*_*c*_ in the low temperature regime, this prediction is also valid at all temperatures in the underdamped regime, where *P*_d_ = 1. Figure 5 illustrates that this theoretical prediction (dashed line) correctly describes the numerical data (full circles), and scales as 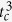 and as *t*_*c*_ in the overdamped and in the underdamped limit, as predicted in Eqs 5 and 8, respectively. In the figure, we also illustrate numerical results for the contribution to the diffusion coefficient of the intra-well jumps (full diamonds), that behaves as predicted in Eqs 6 and 10 in two limits. Thus, the overall diffusion coefficient exhibits a crossover between two linear regimes, as 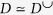 in the overdamped limit, and 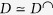 in the underdamped one.

**Figure 6:**
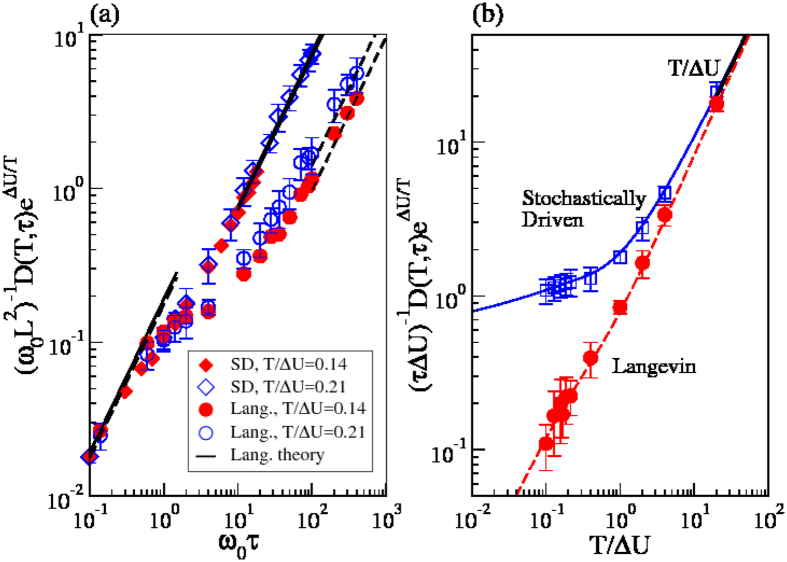
Diffusivities of the Stochastically Driven particle and of the Langevin dynamics, assuming τ = τ_vis_ = t_c_. Panel (a) illustrates the *τ* dependence of the diffusivities, for different temperatures, while panel (b) illustrates the dependence of the diffusivity on the temperature, in the underdamped regime (*τω*_0_ = 10^2^). The full line represents Eq. 8 for the Stochastically Driven dynamics and the dashed line represents Eq. 3.9 of ref. 22 for the Langevin dynamics.

We finally compare the diffusion coefficient of the Stochastically Driven and of the Langevin dynamics, identifying their characteristic timescales, *τ* = *t*_*c*_ = *τ*_vis_. For both dynamics 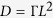 in the overdamped limit. In this limit, the full van Hove distributions actually coincide at all times. In the underdamped low temperature limit, the diffusivity of the Langevin dynamics is 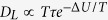
22, while that of the Stochastically Driven particle is given by Eq. 8, 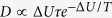. Accordingly, in this limit the Stochastically Driven dynamics is faster than the Langevin one, as illustrated in Fig. 6a. Figure 6b compares the diffusivities of the two dynamics as concern their temperature dependence, in the underdamped regime. The numerical results for the temperature dependence of the Stochastically Driven particle diffusivity are correctly described by our theoretical prediction for 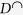 valid at all temperatures (full line), while those of the Langevin dynamics have been predicted in ref. 22. At high temperature, *T* > Δ*U*, the effect of the potential is negligible and the two diffusivities coincide, and scale as *τTe*^−Δ*U/T*^. In the low temperature regime, the Langevin diffusivity does not change temperature dependence, while the diffusivity of the Stochastically Driven particle model only depends on temperature through the Arrhenius factor. The difference in the diffusivities in the underdamped low temperature regime is rationalized considering that the two dynamics are mapped on free hopping dynamics with a different jump rate 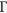, and a different mean square jump length, *λ*^2^. Indeed, on the one side we have already seen that 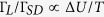. On the other side, in the underdamped limit the mean square size of the jumps of the Langevin dynamics^22^ scales as 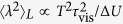, while that of the Stochastically Driven dynamics scales as 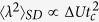, as in Eq. 7, so that 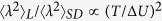. Thus, despite making more frequent irreversible barrier crossing jumps, the Langevin dynamics has a smaller diffusivity as its jumps are much shorter.

## Discussion

We put forward an analytical treatment of the escape rate from a well and of the diffusion coefficient in a periodic potential of a Stochastically Driven particle, considering both the overdamped and the underdamped limits. The particle behaves as a Langevin particle in the overdamped limit. In the underdamped low temperature limit, conversely, with respect to a Langevin particle a Stochastically Driven one has a smaller escape rate, but a larger diffusion coefficient.

Our observations are relevant to describe the dynamics of systems that undergo infrequent collisions with bath particles. Thus, our results could describe chemical reactions occurring at very low pressure, as in this case gas particles seldom collide with the system of interest. Similarly, our results could be relevant to discuss diffusion in amorphous materials at low temperature, when the system can be seen as hopping through different minima of its energy landscape. In this case, the heat bath is provided by the scattering of phonons, whose collision frequency is small at low temperatures^23^.

An interesting feature of this model, which is also observed in a variety of soft-matter and biological systems^9^,^24^,^25^, is the long coexistence of a van Hove distribution with non gaussian tails, and of a mean square displacement linear in time. An important open question ahead concerns the temporal evolution of the van Hove distribution in the underdamped limit, to rationalize how normal diffusion is recovered.

## Methods

### Simulation details

In the Stochastically Driven dynamics, a particle in position *x*_*s*_ = *x(t*_*s*_) that collides with the heat bath acquires a velocity *v*_*s*_ = *v(t*_*s*_) and an energy 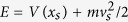. This energy is conserved up to the time *t*_*e*_ = *t*_*s*_ + Δ*t* of the next collision of the particle with the heat bath. At time *t*_*e*_, the particle will be in position *x*_*e*_, the end-point of the jump. In the overdamped and intermediate regime we determine *x*_*e*_ by numerically integrating the equation of motion during a jump, i.e. from the time of the collision *t*_*s*_ to the time *t*_*e*_, using a simple explicit Euler scheme. In the underdamped regime, it is computationally convenient to follow a different approach. Suppose, for instance, that after a collision a particle has enough energy to overcome an energy barrier. In this case the particle will actually traverse many wells, as the jump duration Δ*t* is large. Let Δ*t*_*top*_ the time the particle needs to reach the top of the barrier, and Δ*t*_*cross*_ the time it needs to traverse a well. Starting, without loss of generality, from the first well with positive velocity, the arrival point is conveniently estimated as 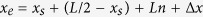, where *n* is the interger part of 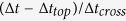, and Δ*x* is the distance the particle moves from the top in a time 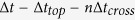. This expression for *x*_*e*_ is convenient as the various quantities can be analytically computed, for our model potential. However, their evaluation requires the evaluation of an elliptic integral, which is time consuming. Thus, this approach is actually convenient only in the underdamped regime. A similar approach can be used when the particle has not enough energy to cross an energy barrier, in which case the particle will perform many oscillations within a well, in the underdamped regime.

The simulation of the Langevin dynamics is more time-consuming that that of the Stochastically Driven dynamics. We have carried it out fixing the integration timestep at 10^−2^* ω*_0_*t*_*c*_.

### Measurement of 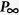 and *P*_d_


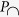
, the probability that a jump crosses a barrier, is easily determined in simulations as the long time limit of the ratio between the number of barrier crossing jumps, *M(t*), and the total number of jumps, *N(t*): 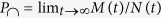. This is the quantity represented by the data points of Fig. 2a. *P*_d_ is the fraction of barrier crossing jumps that are irreversible, i.e. which are followed with equal probability by a forward or by a backward jump. Operatively, this quantity is measured in the simulation by recording the number of forward jumps *f(t*), which are the jumps having the same direction of their predecessor, and the number of backwards jumps, *b(t*). Thus, the total number of barrier crossing jumps is *M(t*) = *f(t*) + *b(t*), and 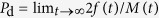.

## Additional Information

**How to cite this article:** Piscitelli, A. and Pica Ciamarra, M. Escape rate and diffusion of a stochastically driven particle. *Sci. Rep.* **7**, 41442; doi: 10.1038/srep41442 (2017).

**Publisher's note:** Springer Nature remains neutral with regard to jurisdictional claims in published maps and institutional affiliations.

## Figures and Tables

**Figure 1:**
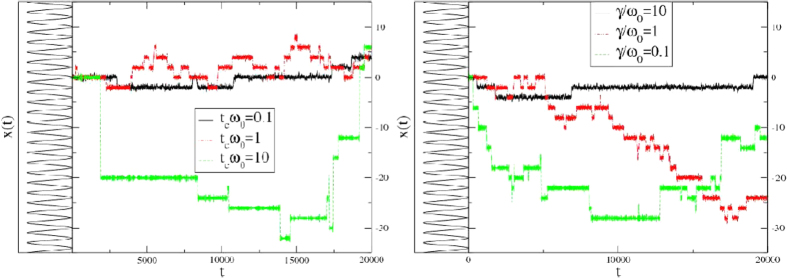
Typical trajectories of the Stochastically Driven particle (left) and of the Brownian particle (right), for ω_0_t_c_ and (γ/ω_0_)^−1^ equal to 10^−1^, 10^0^, 10^1^ at T/ΔU = 0.21.
